# Intestinal Obstruction from an Abscess behind the Posterior Layer of the Peritonæum; Abdominal Section; Recovery

**Published:** 1883-10

**Authors:** Charles T. Parkes

**Affiliations:** Professor of Anatomy, etc., Rush Medical College


### ARTICLE IV.

### INTESTINAL OBSTRUCTION FROM AN ABSCESS BEHIND THE POSTERIOR LAYER OF THE PERITONÆUM—ABDOMINAL SECTION—RECOVERY. By CHARLES T. PARKES, M.D., Professor of Anatomy, etc., Rush Medical College.

The patient, Isaac Meier, aged 21, a clerk by occupation, had never been previously ill. He called at Dr. Stanley's office on the morning of July 11, complaining of pain all over the abdomen, and some nausea. Upon examination, the doctor found the abdomen greatly tympanitic, and regular in contour. There was slight pain on pressure, and the tongue was coated. Temperature 100°, pulse 85. Gave him a pill containing oxalate of cerium, ext. belladonna and carbonate of bismuth. Ordered him to take injections every two hours until he had a passage from the bowels.

The patient had already taken Seidlitz powders without any relief from his constipation or distress. In the evening he sent for Dr. Stanley, who found him in great pain. The injections had had no effect, and the doctor, thinking they had been improperly given, administered a large one, and this also came away without being of any avail. Gave one-fifth of a grain of morphia sulph. hypodermically. The next morning the patient was about the house, feeling much easier; had no movement of the bowels. Again sent for Dr. Stanley in the evening, who found the symptoms the same. Ordered a powder of jalapa and calomel āā gr. viii, to be followed in three hours $\frac{1}{4}$ gr. morphia sulph., if no pain. Called next day. Found no change in symptoms. Bowels unmoved. Requested a consultation, which was granted. After consultation with Dr. Marguerat, the patient was given one drop of croton oil, to be repeated in two hours if the first dose was ineffectual. Morphia to be continued at proper intervals, if suffering pain. The patient continued about the same until the Monday following—six days from the first appearance of his trouble, when he began to vomit stercoraceous matter rather freely and quite often.

The temperature and pulse varied very little from their condition when the patient was first seen until to-day, when the temperature was 97½° and pulse 106 and very feeble. Advised his removal to a hospital.

Dr. Stanley has very kindly furnished me the above history of this interesting case previous to the time when the man was placed in my charge July 17, 1883. I found him with a very rapid and feeble pulse—130, and with a temperature of 100°. The body is bathed with a cold perspiration. There is present a constant hiccoughing and occasional vomiting of a very foul-smelling stercoraceous matter. The abdomen is slightly tender all over, upon palpation, and is only moderately distended. Cannot find any positive evidence of tumor or other sign indicating the point of obstruction, except that the percussion note is not as distinct over the right iliac region as elsewhere. No hernia present.

Having no history of the case at hand, and the patient being too ill to give any, of the commencement and progress of the trouble so far, nor what had been done for him, it was determined to delay any active interference until these points could be ascertained. Meantime, ⅓ of a grain of morphia sulph. was administered hypodermically. The patient was placed in the knee-elbow position, and a large enema of soapsuds was given by hydrostatic pressure. The water was allowed to pass into the bowel until the patient complained of considerable pain. The enema, about three pints in quantity, soon passed away free from fœcal matter or odor, and was unaccompanied with flatus. The diagnosis made was intestinal obstruction, whether intussusception or volvulus undetermined. The patient was told that, in all probability, nothing but abdominal section would enable the obstruction to be relieved. He consented to have the operation performed as soon as his friends were acquainted with the circumstances. They could not be seen before the next morning, July 18. This arrangement was agreed to, partially to gratify this natural wish of the patient, and mainly to try the effects of complete narcotism, together with a repetition of the enema. The evening condition of July 17 was practically the same as that found at the morning examination. The morphia had subdued the pain and partially controlled the hiccoughing, but there was still occasional regurgitation of fœcal matter; it could scarcely be called vomiting. The enemata, given as before, had produced no result, and came away unstained. The pulse was quieter. Morphia hypodermically was ordered to be continued as indications required. The large enemata were discontinued, and small ones, of milk ℥i and whiskey ʒi, given for nourishment; these were retained.

Wednesday, 10 A. M., found the patient evidently worse and failing rapidly, and the friends consenting, operative interference was determined upon.

At 4 P. M. the patient was anæsthetized, Prof. Gunn, and Drs. Reynolds, MacNeil, Stanley and Hanson being present.

An incision four inches long, between the umbilicus and pubes, directly along the linea alba, was carried through the abdominal walls and the peritoneal cavity opened. The intestines first showing in the wound were moderately distended and dark in color. These were pushed aside, and a collapsed fold thereof found low down toward the pelvis. While following this toward the ileocœcal junction, my finger, with slight pressure, went through a thin wall into a cavity, out of which immediately welled up, into the peritoneal cavity, among and around the intestines, and flowed out of the abdominal incision, an immense quantity of horribly offensive and greenish colored pus. The finger was kept in the opening it had produced, and the abscess cavity thoroughly explored. It was behind the posterior layer of the peritonæum, and extended as high as the body of the third lumbar vertebra, and as low as the brim of the pelvis, and into the right iliac fossa half the distance of its width. No fœcal matter nor any other foreign bodies were found in it. When full and distended, the abscess may have caused an acute bend of the ileum, by pressure, just where the small intestine joins the large, sufficient to obliterate the canal of the former. The intestines showed but very little sign of inflammation; were not matted together, and there was no evidence of perforation or inflammation of the appendix vermiformis. In fact, no cause for the obstruction to the intestines other than the abscess was found, after full and complete search.

The opening into the abscess was enlarged with the finger, a rubber tube introduced into it and held in contact with its deepest part. The other end of the tube was attached to a spout in the bottom of a bucket filled with warm water. The bucket was held about two feet above the patient's body, and the cavity of the abscess washed out in all its recesses. The washing was continued until the returning water was perfectly clear. The same plan was pursued in cleansing the peritoneal cavity, fully two gallons of water being used. A soft rubber drainage tube, of large size, was carried to the bottom of the abscess and fastened in the lower angle of the abdominal wound, and this was closed with ten silk sutures. The wound of incision was dressed dry with iodoform and borated cotton, over this some layers of carbolized gauze and a wide bandage, and patient was carried to bed and placed so as to lie with the wound dependent, in order to favor drainage. Instructions were given when he recovered from the anæsthetic to have him maintain that position.

The patient showed evidence of considerable shock, but soon rallied nicely by means of artificial heat and a few hypodermics of whisky. The anæsthetic—ether—acted very kindly, and the patient never vomited subsequent to its administration.

8 P.M.—The patient found entirely free from pain. Reaction established. Pulse 120. Temperature 100°. Given $\frac{1}{4}$ gr. morphia sulph. hyprodermically.

8 A.M. July 19.—The patient has passed a very comfortable night; has no pain; has no vomiting; no hiccoughing. Abdomen not tender. Pulse 100. Temperature 101°. No movement of the bowels.

8 P.M.—Pulse 100. Pemperature 101$\frac{1}{2}$°. The patient is restless, but has no special pain; no vomiting. The dressings are wet with discharge; were changed. The patient has pulled out the drainage tube. He will not give any reason for doing this.

Tried to reintroduce it but failed to do so. The discharge is free in quantity, colorless and odorless. The abdomen is undistended and free from tenderness. The wound does not look well—a reddish hue along the whole length of it. Dressings reapplied as before. A hypodermic injection of morphia sulph., gr. $\frac{1}{3}$, atropia sulph., gr. $\frac{1}{100}$, was given. Has taken milk during the day, retained it and asks for more. Milk to be given in small quantities. Has passed wind per rectum several times during the day.

During the night—4 A.M. July 20th—the patient became delirious, escaped from his attendants, got out of the window, climbed a high fence and went to a neighbor's house, where he was found and brought back to his bed.

At 8 A.M. I found him rational and resting quietly. The dressings were all disarranged and had to be removed Several stitches at the lower part of the wound have been pulled out, probably by the patient's movements during his escapade. The deeper part of the wound seems well united. No union of the integument. The edges looks red and angry. They were supported by strips of rubber plaster. Again tried to introduce the drainage tube but failed. There is but little discharge from the wound. The dressing reapplied.

8 P.M.—Patient's bowels have moved of their own accord this afternoon. No pain. No tenderness. Perfectly rational. Pulse 80. Temperature 99°. No medicine to be given. Continue nourishment.

From this time on the patient progressed rapidly towards recovery. The bowels acted freely and well every day of their own accord. Appetite returned and temperature and pulse became normal.

At the end of a week all stitches were removed from the wound. The lower half gaped widely open. The deeper structures, seemingly, are well united. The muscular tissue is partly exposed. I introduced two sutures some distance away from the edges of the wound and brought it together by tying them over a quill. This plan answered admirably, and healing by granulation progressed rapidly. At the end of the third week—August 7—the patient was discharged well. He has called at my office twice since his discharge. and has returned to his employment.

*Remarks.*—One point in this case seems somewhat noticeable, namely, the absence of any extreme rise of temperature or rapidity of pulse, notwithstanding, there was complete obstruction of the intestines for eight days, with attending tympany and peritoneal inflammation. Still, in my experience even severer trouble with the intestinal tube, is not always accompanied with any remarkable change in these signs of constitutional impression. In one case, coming to mind, where post mortem showed gangrene of several inches of the intestine, neither the temperature nor pulse reached 100.

Other signs than abnormal variation of temperature and pulse must furnish the warrant for surgical interference—these alone do not supply trustworthy indications as to the degree of local difficulty.

Judging from this instance, the presence of pus in the peritonæal cavity is not of itself deleterious. Worse smelling or more offensive pus never came out of an abscess than here bathed the intestines and spread all through the cavity. Still, no further inflammation was lighted up, and that already existing quickly subsided. True, as far as it was possible to estimate, every drop of it was washed out of the cavity and from the surface of the intestines by a good force of warm water and in good quantity. The case certainly supports Mr. Lawson Tait in his advice and practice, to perform abdominal section and wash out the cavity in all cases where accumulation of pus is suspected, no matter what the source or cause.

The difficulty attending the determination of the cause of intestinal obstruction, previous to abdominal section, is recognized by all surgeons, and is used, in part, as an argument against the operation by most surgical writers. Omniscience alone could have guessed the cause of the obstruction in this case. Pain, vomiting and hiccoughing were relieved immediately by the evacuation of the abscess, and never returned. The passage of wind from the bowel a few hours after the operation, suggested the restoration of patulency to the intestinal tube, and the free discharge of fœcal matter inside of thirty-six hours, made the suggestion a reality. I suspect that the slight attack of delirium after the re-dressing of the wound was caused by the absorption of iodoform.

Quite a number of cases of intestinal obstruction seen in my own practice, as well as in the hands of other medical men, which have rapidly passed on to death under the ordinary methods of treating them, have for some time convinced me, that no case of this kind ought ever to be allowed to go without the chance of recovery after abdominal section. Many cases may die even with its reasonable help, but I am quite as sure, many lives would be saved by the adoption of a rule to operate.

